# Edge Integration of Artificial Intelligence into Wireless Smart Sensor Platforms for Railroad Bridge Impact Detection

**DOI:** 10.3390/s24175633

**Published:** 2024-08-30

**Authors:** Omobolaji Lawal, Shaik Althaf Veluthedath Shajihan, Kirill Mechitov, Billie F. Spencer

**Affiliations:** Department of Civil and Environmental Engineering, University of Illinois, 205 N. Matthews Ave, Urbana, IL 61801, USA; oglawal2@illinois.edu (O.L.); sav4@illinois.edu (S.A.V.S.); mechitov@illinois.edu (K.M.)

**Keywords:** impact detection, railroad bridge, structural health monitoring, edge implementation, artificial intelligence, machine learning

## Abstract

Of the 100,000 railroad bridges in the United States, 50% are over 100 years old. Many of these bridges do not meet the minimum vertical clearance standards, making them susceptible to impact from over-height vehicles. The impact can cause structural damage and unwanted disruption to railroad bridge services; rapid notification of the railroad authorities is crucial to ensure that the bridges are safe for continued use and to affect timely repairs. Therefore, researchers have developed approaches to identify these impacts on railroad bridges. Some recent approaches use machine learning to more effectively identify impacts from the sensor data. Typically, the collected sensor data are transmitted to a central location for processing. However, the challenge with this centralized approach is that the transfer of data to a central location can take considerable time, which is undesirable for time-sensitive events, like impact detection, that require a rapid assessment and response to potential damage. To address the challenges posed by the centralized approach, this study develops a framework for edge implementation of machine-learning predictions on wireless smart sensors. Wireless sensors are used because of their ease of installation and lower costs compared to their wired counterparts. The framework is implemented on the Xnode wireless smart sensor platform, thus bringing artificial intelligence models directly to the sensor nodes and eliminating the need to transfer data to a central location for processing. This framework is demonstrated using data obtained from events on a railroad bridge near Chicago; results illustrate the efficacy of the proposed edge computing framework for such time-sensitive structural health monitoring applications.

## 1. Introduction

Railroads play a critical role in the United States transportation sector, serving as one of the primary means of freight transportation. According to the United States Bureau of Transportation Statistics, railroads were responsible for moving 18.5 percent (by ton-miles) of the nation’s freight, worth USD 403 billion, in 2023 [1]. Bridges form a vital part of the rail freight network. Half of the 100,000 railroad bridges in the United States are over 100 years old and were constructed when the minimum vertical clearance requirements were lower than the current standards, thus making them prone to impact by over-height vehicles [2,3]. These impacts on railroad bridges can exceed the bridge design impact load and can lead to structural damage as well as service disruptions, resulting in danger to public safety, disruption of traffic, and potentially significant loss of revenue for railroad owners [4].

While numerous studies have been conducted that emphasize the gravity of the railroad bridge impact issue in the United States [5,6,7], the railroad bridge impact problem is not limited to the United States. Results from a survey conducted by the World Road Association show that various railroad bridge owners across the globe consider bridge impacts as a major issue [8]. NetworkRail in the United Kingdom reported that there were 1624 railroad bridge impacts between April 2020 and March 2021 across their network [9]. In a study conducted by Coleman et al. [10], they reported that the United Kingdom rail network experiences around 2000 railroad bridge impacts yearly, which results in the loss of approximately GBP 23 million for the rail authorities. Similar issues are found in New Zealand, where railroad bridge impacts cause significant delays and cost KiwiRail an average of around NZD 650,000 per year between 2010 and 2014 [11]. The above data show that bridge impacts are a significant problem worldwide with huge cost implications for railroad owners.

While major full-on impacts are relatively rare, minor impacts, such as when vehicles scrape underneath bridges, can occur frequently. Because such scrapes can compromise the structural integrity of railroad bridges and often go unnoticed, detection is important. The most common approach for impact detection involves the monitoring and instrumentation of bridges with accelerometers; impacts are assumed to occur when the vibration levels exceed a predetermined threshold. These monitoring systems generally make use of wired sensors that have been widely adopted in practice for structural health monitoring (SHM) [12,13,14]. However, these wired SHM systems often require substantial labor and are costly to deploy and maintain.

Wireless smart sensor (WSS) technologies have emerged as promising alternatives to traditional wired sensor systems in SHM applications, offering advantages such as easier installation and reduced maintenance costs [15]. WSSs are equipped with on-board processing capabilities and wireless communication, which enable them to provide rich information [16,17,18], allowing the assessment of a structure’s condition to be conducted more autonomously, efficiently, and on demand. To this end, Fu et al. [19] developed a wireless monitoring system for detecting impact events for long-span highway bridges. Moreover, the system employed predetermined thresholds to identify impacts. Although a threshold-based approach can identify major impacts that result in sufficiently large vibration, the more common railroad bridge impacts are often difficult to distinguish from train crossings. Therefore, the use of a threshold-based approach is prone to misclassification of events and unreliable for impact detection.

To address this issue, researchers have proposed the use of machine learning for railroad bridge event classification and impact detection based on acceleration responses obtained from WSSs [4,20,21]. These studies utilize cloud computing, which involves first transferring the data remotely to a centralized location (i.e., the cloud) where the machine-learning models are applied. The results show that the use of machine learning produces more accurate outcomes in terms of correctly classifying railroad bridge events and identifying impacts. Hoang et al. [18] developed a cloud-based data retrieval and visualization framework for railroad bridges. Dang et al. [22] proposed a cloud-based digital twin framework for SHM and validated the approach on a railroad bridge. However, these studies may not be effective for applications that require real-time or near-real-time inferences. The challenge arises due to the significant time required to transfer the entire dataset from the sensor nodes to a central location over low-bandwidth wireless connections and potential wait times due to the queued data from a previous event. Additionally, the target railroad bridges can be in areas with poor connectivity, resulting in intermittent delays in uploading data. Therefore, to meet the demand of real-time or near real-time decision-making, the computation needs to be performed on the WSS.

Edge computing paradigms seek to deploy computational resources at or near the location where the data are acquired, thus reducing or eliminating the need for large amounts of data to be collected at a centralized hub [23]. Some researchers have implemented edge computing on WSSs in SHM applications. For example, Mondal et al. [24] proposed a theoretical framework for anomaly detection using edge computing. Hoang et al. [18] proposed an approach for on-board reference free displacement estimation from a measured acceleration that was implemented on the Xnode WSS platform [25,26,27,28]. V. Shajihan et al. [29] implemented edge computing for vision-based displacement estimation of in-service railroad bridges. Despite these developments, current edge computing hardware and software implementations on typical WSSs lack the necessary resources to efficiently support making machine-learning predictions using measured data that will facilitate real-time or near real-time decision-making.

In this paper, a new artificial intelligence (AI)-enabled WSS framework is proposed that will facilitate machine learning at the edge. The proposed framework integrates the capability of the OpenMV H7 Plus module [30] to implement machine-learning models directly with the Xnode WSS platform, leveraging its wireless sensing and communication abilities. Rapid notification of impacts enables a quick response and minimizes risk to the public after railroad bridge impacts. Subsequently, the efficiency of the framework is illustrated using field-collected data from railroad bridge events. The remainder of the paper is organized as follows: Section 2 discusses the devices required to create a framework for an AI-enabled WSS and the integration of an edge device and a next-generation WSS platform. Section 3 introduces the example application used to validate the developed framework. Section 4 demonstrates the efficiency of the proposed approach using railroad bridge field data. The developed framework is shown to be highly flexible, allowing it to be applied to other SHM tasks that require making machine-learning predictions on a wireless sensor platform.

## 2. AI-Enabled Wireless Smart Sensor

This section presents an overview of the hardware for the proposed AI-enabled WSS, which integrates the Xnode platform [25,26,27,28] with the OpenMV H7 Plus module [30]. Following a brief description of the Xnode platform and its limitations with regard to implementing AI models, the features of the OpenMV H7 Plus module are discussed. The framework proposed for enabling AI capabilities on the Xnode is then discussed.

### 2.1. Xnode

**Figure 1:**
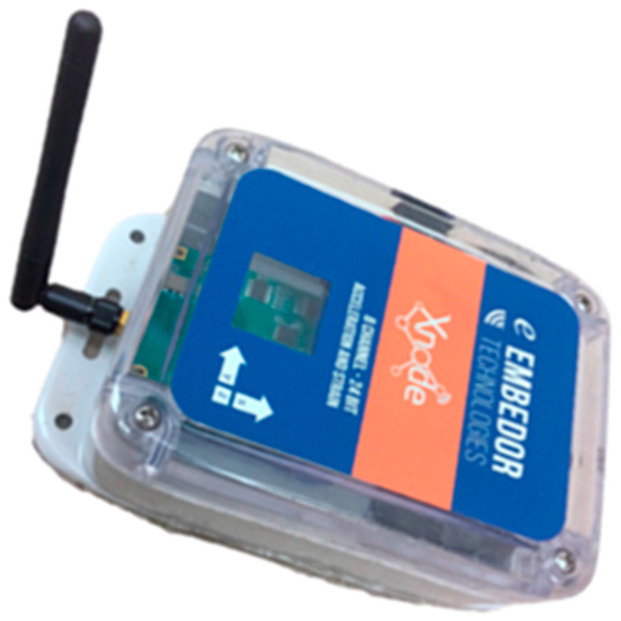
Enclosed Xnode WSS.

The Xnode is a next-generation WSS platform that has a 24-bit analog-to-digital converter, a built-in three-axis accelerometer, eight sensing channels, solar energy harvesting, etc. The Xnode firmware is based on the Illinois Structural Health Monitoring Project Services Toolsuite [31], which is a software service library for long-term SHM of civil infrastructure. The developments made by Fu et al. [17] ensure that the Xnode is capable of event-triggered sensing to capture sudden events while minimizing power consumption. This feature makes the Xnode suitable for the task of detecting the occurrence of sudden events. The Xnode’s modular architecture offers the flexibility for possible connections to other external sensors [32] and possesses long-range wireless communications as well as cloud storage for retrieved data [18]. The Xnode has been found to be effective for several SHM applications (e.g., cable tension monitoring [33] and high-fidelity sensing [27,34]). Figure 1 shows an enclosed Xnode WSS. Despite these capabilities, the Xnode lacks the necessary resources to implement machine-learning tasks.

WSS platforms such as the Xnode used in this research do not have machine-learning libraries available that would allow for direct implementation of machine-learning tasks at the edge of these devices. Furthermore, the Xnode software library was developed using the C programming language, and implementing efficient low-level code for machine learning on an embedded device in C is complex. Finally, to support recent advancements in neural network models, a more powerful processor than the Xnode’s Arm Cortex M4F is required to make inferences and eventually train machine-learning models on-board. To this end, the open-source, compact, energy-efficient OpenMV H7 Plus [30] module is chosen to be integrated into the Xnode WSS platform.

### 2.2. OpenMV H7 Plus

**Figure 2:**
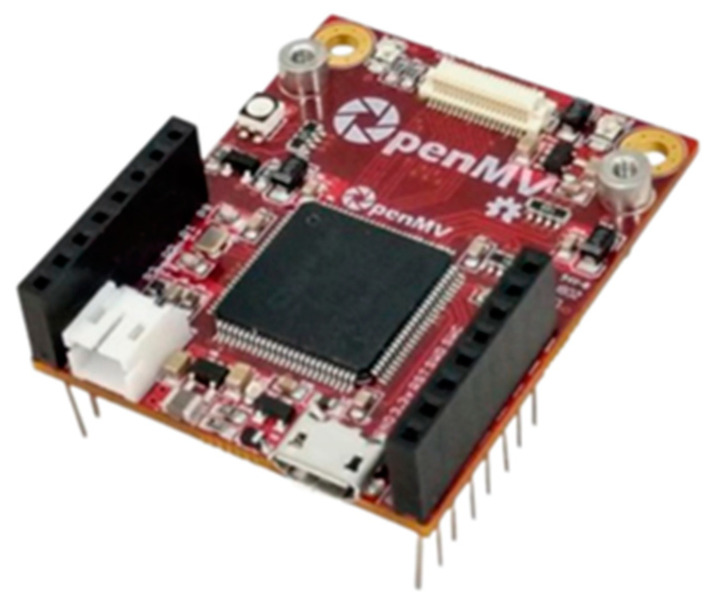
OpenMV H7 Plus microcontroller board [40].

OpenMV is an embedded systems platform with microcontroller-based boards tailored for the rapid development and deployment of machine-learning applications. The OpenMV H7 Plus module, herein referred to as the OpenMV module, is selected for this research and uses a Micropython [35] interface, which is desirable due to the abundance of available open-source Python libraries, therefore facilitating accessibility and ease of development. In particular, an array of open-source machine-learning libraries is offered, allowing for the implementation of a wide range of machine-learning applications. The module is compact, energy-efficient, and has been widely applied in various domains, including robotics [36], Internet of Things (IoT) devices [37], drone technology [38], etc. The OpenMV module has also been used for SHM applications, such as the displacement monitoring of railroad bridges [29] and concrete crack detection [39]. The aforementioned features make the OpenMV module suitable for the edge implementation of machine-learning algorithms for SHM tasks. Table 1 summarizes the technical specifications of the OpenMV H7 Plus microcontroller board, while Figure 2 shows an image of the OpenMV board. The integration of the Xnode and OpenMV platforms is discussed in the next subsection.

### 2.3. Framework for AI-Enabled Wireless Smart Sensor

**Figure 3:**
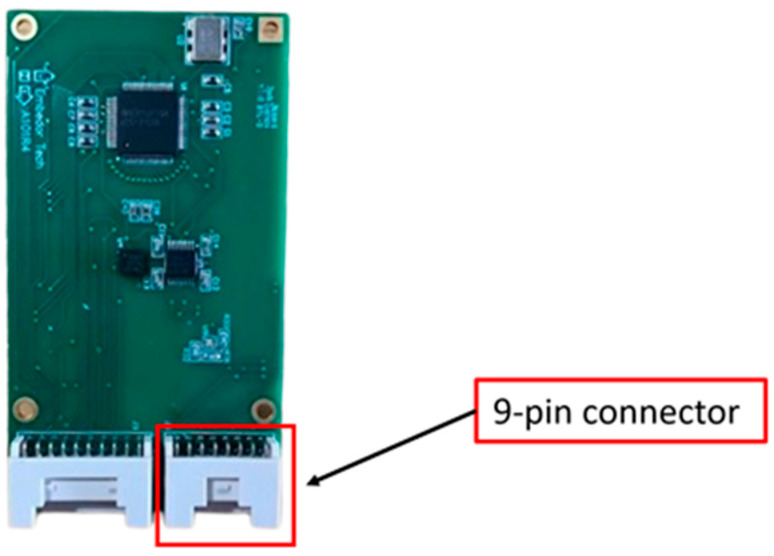
Xnode sensor board.

In this study, an edge device, the OpenMV module, is integrated with the Xnode to enable machine-learning predictions on a WSS platform. The Xnode’s sensor board, A101-R4 (see Figure 3), is designed to allow connections with external sensors. The connection between the OpenMV module and the Xnode is established using a 9-pin connector. The connector provides power (3.3 V), ground, communication, and general-purpose control pins from the Xnode to the external device [18].

The OpenMV module communicates with the Xnode using the Universal Asynchronous Receiver/Transmitter (UART) serial interface. The UART serial interface operates at 115.2 kbps using eight bits per character and one bit for the stop bit. The software development to enable the integration of the OpenMV module with the Xnode platform is challenging because accurate real-time task handling and effective memory management are required for reliable edge computing on resource-constrained devices. The Xnode software framework utilizes a real-time operating system (FreeRTOS), which allows for precise control over task scheduling and memory allocation [26,27]. To that end, the software configuration of the Xnode’s FreeRTOS is performed in the “RemoteSensing” application [26] to schedule data acquisition tasks and manage the communication with the OpenMV module. In this application, the Xnode is employed as the control device, which sends commands to communicate with the OpenMV module using the UART interface. The OpenMV module software integration is implemented using Micropython to handle communication with the Xnode, load machine-learning models, and run inference.

The operational workflow of the AI-enabled WSS begins with the detection of an event by the ADXL362 trigger accelerometer [17]. Upon the occurrence of an event, the ADXL362 wakes up the Xnode to start sensing. Note that an event is detected when the vibration level exceeds a predetermined threshold. Simultaneously, the OpenMV module will be powered on. Because the Xnode is the controlling device, the OpenMV module is programmed to wait for a “start” command from the Xnode before executing any further tasks. Once the sensing task is completed (i.e., the vibration level drops below the threshold) and the Xnode has acquired all sensing data, the “start” command is sent to the OpenMV module, and the sensing data are sent via the UART interface. Upon receiving the “start” command, the OpenMV module is coded to check the UART interface and read any data received, four bytes at a time, until all the sent data have been stored in arrays on the OpenMV module for on-board processing. After storing all the sensing data, the OpenMV module loads a pre-trained machine-learning model from its memory.

**Figure 4:**
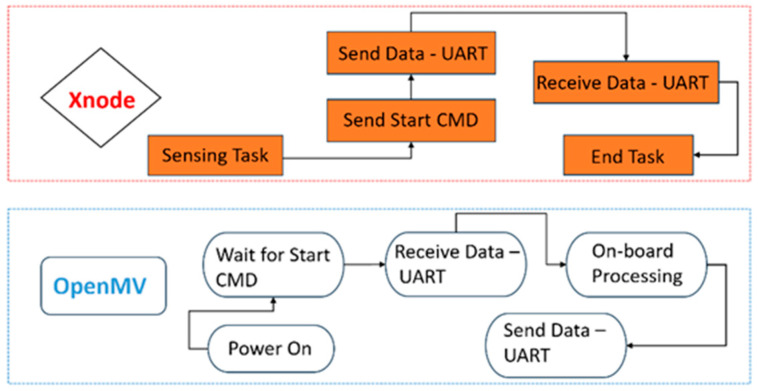
Implemented software tasks for edge implementation.

**Figure 5:**
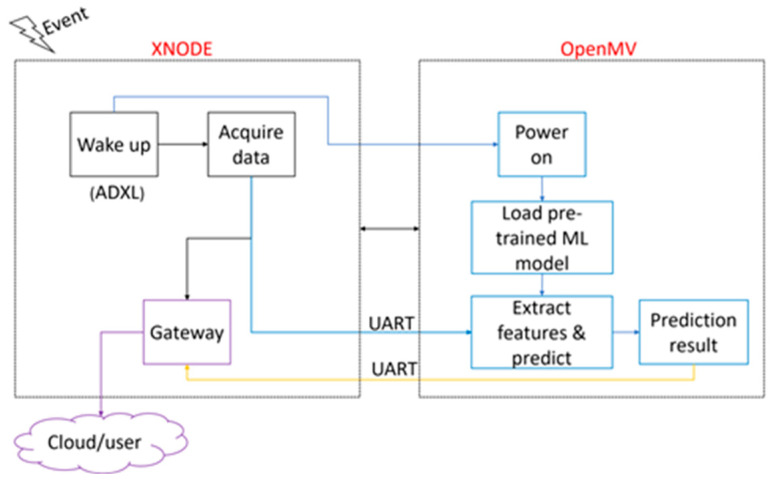
Operational framework for edge implementation.

While the OpenMV module’s processor allows the execution of complex machine-learning models, the maximum model complexity is limited by the available memory and processing speed. In this study, the artificial neural network (ANN) model used for railroad bridge impact detection was designed to fit within the OpenMV module’s memory constraints, as its in-memory size is only 36 KB (OpenMV H7 plus features 32 MB RAM). The ANN was trained offline using a large dataset collected by the Xnode from multiple railroad bridge events. Note that the framework proposed in this study requires pre-trained machine-learning models. Offline pre-training of machine-learning models is typically performed using a personal computer or a cloud-based platform. The training process involves collecting a dataset, preprocessing the data, and then fitting a selected machine-learning model into the data. In this study, the ANN model was trained using the Keras Application Program Interface (API), which provides a wide range of libraries for building and optimizing machine-learning models. After training, the model is quantized to reduce the memory footprint and converted to a Tensorflow Lite format for deployment on the OpenMV module. Tensorflow Lite is a library designed to enable machine-learning models to run on edge devices [41]. The quantization process involves reducing the precision of the model’s parameters without significant loss in accuracy. For inference, features are extracted from the received data, and a prediction is made on the extracted features by passing them into the loaded model. The results from the on-board processing are sent back to the Xnode via the UART interface. After sending the sensing data, the Xnode checks the UART interface to see if any new data have been sent by the OpenMV module. Once the result is received by the Xnode, the task is ended, and the results are uploaded to the cloud. Notifications may also be immediately sent to end users. Figure 4 shows the software tasks implemented on both devices to ensure the integration of the OpenMV module and the Xnode, while Figure 5 shows the operational framework for this task.

## 3. Railroad Bridge Impact Detection—Event Classification

The railroad bridge event classification algorithm proposed by Lawal et al. [21] is implemented on the OpenMV module for this research. For the convenience of the reader, a concise summary of the approach is described in this section.

**Figure 6:**
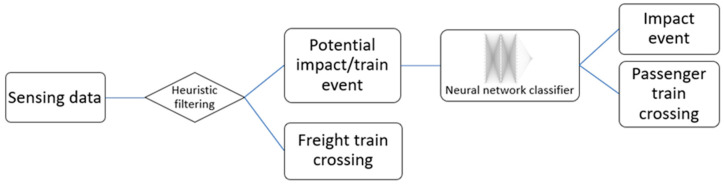
Algorithm for event classification.

A schematic of the event classification algorithm is shown in Figure 6. Because the features considered (see Lawal et al. [21]) are often similar when extracted from freight train crossings and bridge impacts and the fact that freight train events are much more frequent than other event types, a heuristic approach is first used to identify freight trains. This heuristic approach is implemented using an event duration threshold, which is defined based on the typical known length of local freight trains. If the duration of an event exceeds the defined threshold, the event is automatically classified as a freight train event without the need for further analysis. For shorter events, further analysis is still required, as passenger trains can have a duration similar to bridge impact events. Thus, a neural network classifier is developed to delineate between bridge impacts and passenger train crossings. A neural network approach is chosen as a preliminary analysis, and the threshold-based approach or a simple signal processing algorithm is prone to event misclassification. The reduction in false positives to the barest minimum is one of the important goals of this implementation, which is to prevent mobilizing an inspection crew for false alarms. Note that the decision to first use a heuristic approach to identify freight train events helps to address the bias problem in training the neural network, as most of the recorded events correspond to freight train crossings.

For the neural network classifier, a fully connected neural network was implemented comprising two hidden layers, with each layer comprising fifteen nodes. The input layer accepts carefully selected features extracted from the Xnode’s accelerometer data, namely the peak absolute acceleration, the two most dominant frequencies obtained from the Fast Fourier Transform, center of mass as denoted by Sitton et al. [20], and spectral energy. These five features are extracted in each of the three acceleration directions, resulting in a total of fifteen inputs into the ANN. The rectified linear unit (ReLU) activation function is applied to the hidden layers, introducing non-linearity and enabling the network to learn complex patterns. The network was designed for a binary classification problem, producing either 0 or 1 as its output corresponding to the two categories of interest: passenger train crossings and bridge impact events, respectively. A sigmoid activation function is applied to the output layer. Note that all the impact data considered in this work pertain to minor impact events. The construction of the model is executed using the Keras Sequential API in Python.

This algorithm was implemented on the OpenMV in the Xnode. Results will be presented and discussed in the next section. For more details regarding this event classification algorithm, see Lawal et al. [21].

## 4. Results

Because an appropriate railroad bridge was available for field testing, the proposed edge framework for railroad bridge event classification and impact detection is validated using the data previously collected. First, the data and the testing approach are described. Then, the proposed approach is shown to effectively identify impact events and distinguish them from train crossings. Finally, the broader implications and future directions of the research are discussed.

### 4.1. Railroad Bridge Field Data

**Figure 7:**
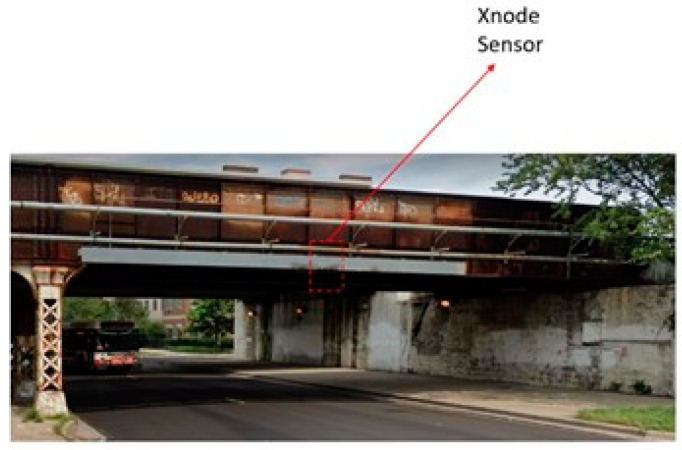
Low-clearance railroad bridge instrumented with the Xnode.

Data from a railroad bridge (see Figure 7) in northern Illinois was collected over 44 weeks in 2021–2022 using the Xnode wireless sensor. During this period, 20 freight trains and five passenger trains crossings occurred in a typical day; additionally, the bridge was impacted by over-height vehicles 105 times during this period, as detailed in Lawal et al. [21]. For all events, a 100 Hz sampling rate was used with the ADXL362 accelerometer, while data from the Xnode’s high fidelity accelerometer, the ST Micro LIS344ALH, are sampled at 1 kHz before being downsampled to 100 Hz. Two Xnode WSSs were mounted on crash beams and placed near the midspan of the bridge, one on each side. Note that for short single-span bridges like the one used in this study, the sensor measurements (i.e., obtained acceleration responses) are expected to be similar regardless of the sensor location; thus, the Xnode is able to capture the significant modes contributing to the overall response. The collected dataset encompasses a wide range of events with varying acceleration magnitudes, providing a comprehensive representation of the events that occurred on the bridge over nearly a year. The consistent sampling rate of 100 Hz for all events ensures uniformity in data representation, facilitating easier comparison between different events and simplifying machine-learning implementations. Finally, the high volume of recorded events provides sufficient data for training a robust model.

### 4.2. Neural Network Training and Implementation

The training process is a critical step in ensuring that the ANN classifier can effectively distinguish ambiguous railroad bridge events. The process ranges from data preparation to model optimization to ensure the ANN’s accuracy and robustness. The first step of preparing the collected dataset involved labeling events as either train crossings or bridge impacts with the help of bridge engineers. The features mentioned in Section 3 are extracted from the collected dataset and used to train the Keras-based ANN model. To ensure the robustness of the trained model, an equal number of passenger train crossings and bridge impact events are included in the training dataset, totaling 210 events. The dataset is randomly split into 70% training and 30% testing data, with 20% of the training data held out as a validation set. Before training the ANN model, the input features are standardized by removing the mean and scaling unit variance for them. This preprocessing step is performed only on the training data; the obtained parameters are used to transform the test data accordingly. This standardization ensures that all features contribute appropriately to the learning process, regardless of their original scales. The ANN model is implemented using the Keras library. The loss function minimized during training is binary cross-entropy. The Adam optimizer is used to update the network’s weights and biases to minimize the loss function. The model architecture consists of two hidden layers, each with fifteen nodes. The learning rate is set to 0.001, and the number of training epochs is determined through a trial-and-error approach and ultimately set to 200.

**Figure 8:**
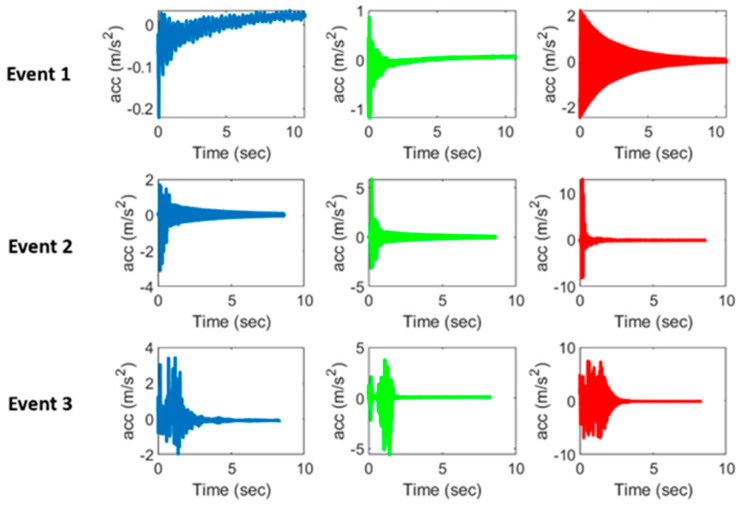
Acceleration record of three impact events in longitudinal (blue), vertical (green) and lateral (red) directions.

**Figure 9:**
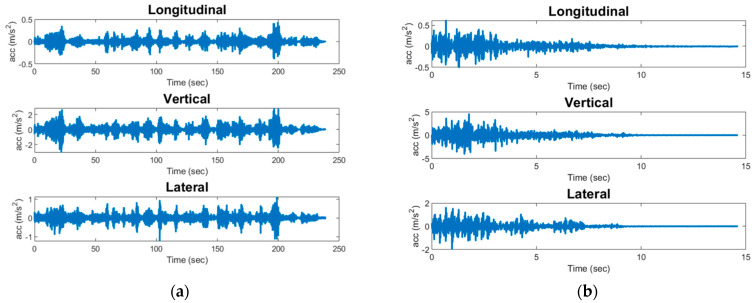
Typical data collected using Xnode for train crossings: (**a**) freight train; (**b**) passenger train.

The trained neural network is saved onto the SD card of the OpenMV module for edge implementation. While the model developed for event classification is lightweight enough to be deployed at the edge of the OpenMV device (37 KB in size), the model is converted to a TensorFlow Lite format to optimize performance on an edge device. The generated TensorFlow Lite model is 4 KB in size. The original lightweight Keras model is converted to TensorFlow Lite using the TensorFlow Lite converter API in Python. The implementation is programmed in Micropython to manage loading the model into the OpenMV module’s memory, feature extraction, and inference execution. For the purpose of this study, pre-recorded events are used to demonstrate the capability of deploying the developed event classification system at the edge of WSSs. Some of the impacts are shown in Figure 8, demonstrating the variation in magnitude and signal characteristics of recorded impacts. Examples of typical passenger and freight train crossings are shown in Figure 9. In this implementation, the acceleration data for these events are saved on the Xnode SD card as a text file to allow efficient reading into the Xnode memory. The data are then sent to the OpenMV module via UART for on-board feature extraction and event classification. Upon receiving the data from the Xnode, the OpenMV module loads the machine-learning model into its memory from the SD card. Features are then extracted from the test signals and are used as input into the pre-trained machine-learning model to run inference and receive the predicted output. Different events are selected because they show the variety in the characteristics of recorded impact signals, including variations in amplitude, duration, and shape of the signals. The primary goal of this implementation is simply to demonstrate the capability of deploying the developed event classification system at the edge of WSSs, with a focus on the data transfer between the Xnode and OpenMV module. The successful transfer and processing of the pre-recorded event data would validate the effectiveness of the proposed edge-deployed event classification algorithm for railroad bridge impact detection.

### 4.3. Results

**Figure 10:**
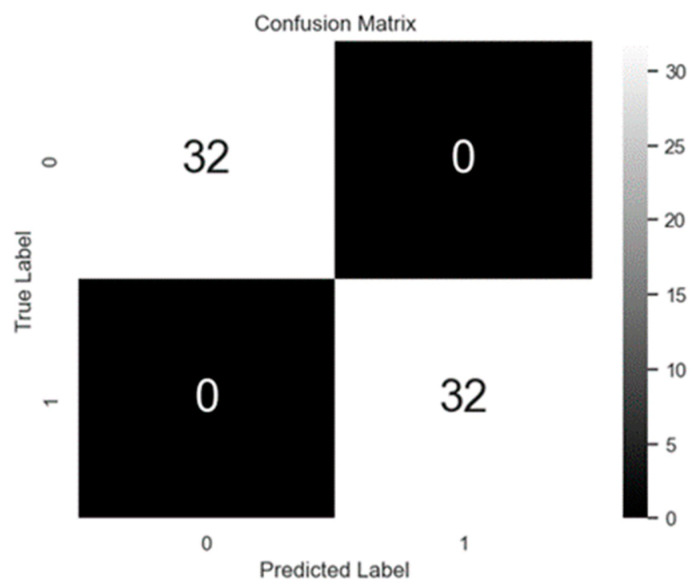
Confusion Matrix for test examples (0 = train crossing, 1 = bridge impact).

Figure 10 presents a confusion matrix of the classification results achieved from running the developed ANN classifier on the test dataset, which comprises 64 events. As mentioned in Lawal et al. [21], the ANN classifier correctly identified all instances of both impact events and train crossings, thus achieving perfect classification accuracy for the examples considered. The classification results highlight the model’s effectiveness in accurately detecting impact events while minimizing false alarms. The results also highlight the advantage of considering all three acceleration directions as the classification accuracy reduces to 87% when only lateral direction is considered and 93% when only both lateral and vertical directions are considered. This high level of performance suggests that the developed system offers reliable impact detection and event classification capabilities that are valuable for practical applications.

To further evaluate the model’s robustness, its performance was validated using a 10-fold cross-validation technique [42]. The cross-validation technique splits the dataset into 10 equal subsets. In each iteration, one subset serves as the test data, while the model is trained on the remaining nine subsets. This process is repeated 10 times, with each subset taking a turn as the test data. The average accuracy achieved across the 10 distinct models generated during the cross-validation process was 98.67%. This result indicates a high level of performance and robustness for the developed model.

The model’s performance across various event types and acceleration magnitudes is critical, given that impacts can occur with different intensities and signal characteristics (for example, the typical peak lateral frequency of the observed impacts varied from 3 to 15 Hz). Table 2 presents the peak accelerations in all three directions (longitudinal, vertical, and lateral) for typical events considered, along with their classification results. These examples were chosen to show that the model successfully identified impact events that span a wide range of peak acceleration. Additionally, some train events have peak accelerations similar in magnitude to certain impact events (e.g., Events 3 and 9), further demonstrating the unreliability of using a simple threshold-based approach for impact detection, as was mentioned in Lawal et al. [21]. In summary, these results highlight the model’s robustness in accurately classifying impacts of different magnitudes and show the importance of a robust classification approach for distinguishing between train crossings and impact events.

**Figure 11:**
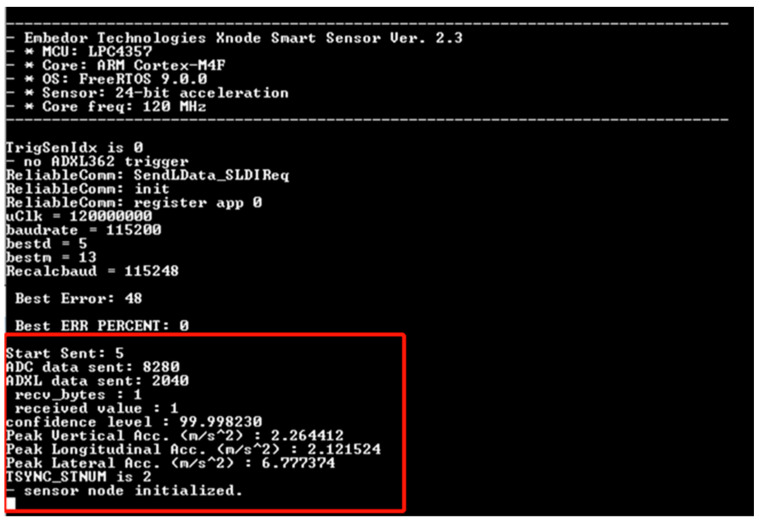
Screenshot of Xnode terminal output after running inference on a test event.

Figure 11 shows an example of the output from running the inference at the edge of the proposed system, including the sending and receiving of data. The inference was run on the Event 1 (top row) signals shown in Figure 8. The model classified the signal as an impact event. Note that, to efficiently utilize memory resources, only a single byte is sent back from the OpenMV module to the Xnode as the result-1 if impact and 0 if not impact (instead of the full string of the result, i.e., “impact”). Thus, for this test example, the Xnode received a value of “1”. Furthermore, the confidence level, which represents the probability assigned by the neural network model that an event is indeed an impact, is also shown. Finally, the model also outputs the peak acceleration in all three directions to help bridge engineers understand the magnitudes of detected impact events. Note that the peak acceleration is determined using both the high fidelity and ADXL362 accelerometer data. Finally, the average inference time from the moment data are sent from the OpenMV module until the prediction is sent back to the Xnode is 0.93 milliseconds.

Current draw is an important factor to be considered in wireless sensor applications. The Xnode with the OpenMV module draws approximately 190 mA during sensing, representing only a 12% increase over the baseline Xnode power consumption. This modest increase in the current draw is particularly favorable considering the significant additional capabilities that the OpenMV module provides for edge computing and machine-learning tasks. The relatively low power consumption of the integrated system demonstrates its efficiency and suitability for long-term deployment in wireless SHM applications.

## 5. Conclusions

In this paper, an AI-based framework for railroad bridge impact detection was developed using an edge implementation on wireless smart sensors. First, the hardware for the AI-enabled platform was developed by integrating the OpenMV H7 plus module with the Xnode. The framework was validated by implementing a railroad bridge impact detection system at the edge of the Xnode WSS platform. The results demonstrated efficient communication between the OpenMV module and Xnode devices. The event classification algorithm correctly classified all events and identified all bridge impacts with a high level of confidence. The event classification algorithm not only accurately predicts impacts but also outputs the maximum acceleration in all three directions. To efficiently utilize memory resources, the classification results are encoded into a single byte before being sent back to the Xnode.

Future research will explore the capabilities of the OpenMV module’s processor to allow for on-board training of machine-learning models, in addition to using pre-trained models, as was used in the application shown in Section 4.2. On-board training allows the finetuning of pre-trained models when new data become available, making the system more robust and adaptable over time. This capability is particularly valuable as environmental conditions or structural characteristics may change over time, requiring periodic model updates to maintain accuracy. The ability to carry out on-board training also allows for using transfer learning. Transfer learning refers to reusing knowledge learned from one task in another related task. For example, the neural network trained in this work could be adapted for detecting events on highway bridges without the need for extensive data collection. Note that this on-board training can be unsupervised by assuming all new instances to be trained since train crossings occur far more frequently than impact events. However, also note that the impacts in this study include minor impacts that occur more often (once every 1–2 weeks for the monitored bridge) than full-on major impacts. The benefit of this process will be the improvement of the model in identifying train crossings. However, a challenge arises in the potential training on false positive instances during actual bridge impacts, which could affect the model’s accuracy. Lastly, the classification of impacts into different severity categories will be included in a future study.

Finally, while the implementation in this work focuses on railroad bridge impact detection and event classification, the broader implication of this work is that machine-learning applications for SHM are enabled on the Xnode WSS device.

## Figures and Tables

**Table 1:** Technical specifications of OpenMV H7 Plus [30].

Specification	OpenMV H7 Plus
Power	240 mA @ 3.3 V
Microprocessor	Arm Cortex-M7
Clock speed	480 MHz
RAM	32 MB
Programming	Micropython
Open source	Yes
Commercially available	Yes

**Table 2:** Summary results from selected test events.

Event Number	Peak Longitudinal Acceleration (m/s^2^)	Peak Vertical Acceleration (m/s^2^)	Peak Lateral Acceleration (m/s^2^)	Classification Result
1	2.1215	2.2644	6.7773	Impact
2	0.6733	0.8783	1.9868	Impact
3	2.267	8.1734	9.6672	Impact
4	15.1512	19.7129	30.8948	Impact
5	0.169	1.7734	1.664	Train
6	20.4395	29.67	33.1432	Impact
7	0.4972	4.4067	3.7558	Train
8	0.629	5.4063	3.0172	Train
9	2.3407	9.5216	6.1671	Train
10	0.5296	4.8359	3.7874	Train

## Data Availability

Data are contained within the article.
